# Understanding the decay of proteins: A method to study time dependent response of pM concentration of insulin at microwave frequencies

**DOI:** 10.1016/j.mex.2016.11.004

**Published:** 2016-12-15

**Authors:** Ritika Verma, K.S. Daya

**Affiliations:** Microwave Physics Lab Department of Physics & Computer Science Faculty of Science, Dayalbagh Educational Institute, Dayalbagh, Agra 282 005, India

**Keywords:** Insulin, Buffer solution, Whispering gallery mode, Dielectric resonator

## Abstract

Bio-molecule when isolated from its natural ecological condition is subjected to rapid decay. This decay leads to change in polarization and permittivity of molecule. This study presents an experimental analysis of the decay pattern of pM concentration of insulin using whispering gallery mode (WGM) dielectric resonator (DR) method. Analysis is carried out by comparing the permittivity, relaxation time and time delay for three days. It is observed that different pM concentrations of insulin solutions start to decay after 24 h at 5°C. Salient features of the present method are:

• This method presents time dependent analysis to determine the activity of protein solution by measurement of permittivity, relaxation time and time delay.
• In the present paper activity of pM concentration of Insulin in buffer solution is tested for three days.
• This method is a general method and can be a fundamental basis to test the activity of bio-molecules in solution.

## Graphical abstract

**Figure:**
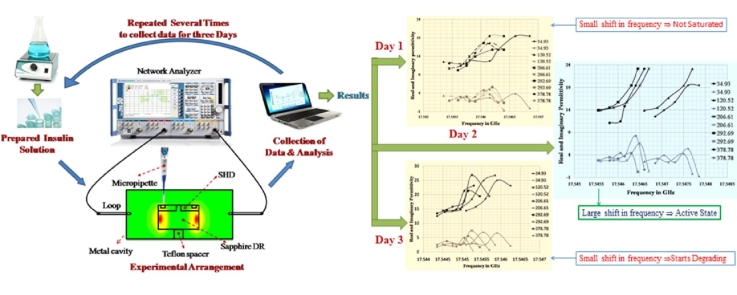

**Table:** Specifications Table

Subject area	*Materials Science*
More specific subject area	*Microwave Bio-sensing*
Method name	*WGM DR method for time dependent activity analysis of Insulin in buffer solution*
Name and reference of original method	*Microwave WGM DR sensing method (Ref.**[11]**)*
Resource availability	*Not necessary*

## Method details

Response of biomolecules in buffer is central in understanding the behaviour of biological systems in their native ecological conditions [1], [2], [3], [4], [5], because most of the biological systems such as proteins are active in aqua medium, and they are found to closely mimic their natural behaviour in a buffer medium *in vivo*. Due to this protein solutions are prepared in buffer at high concentration preferably greater than 1 mg/ml for storage. Below this concentration protein degradation becomes comparatively faster [6]. One such important protein is insulin, which plays primary role in controlling human metabolism and is found in picomolar (pM) concentration in human blood. There are many reports on quantification of insulin [7], [8], [9], [10] but very low concentration of insulin makes such studies very challenging. To study the biological activity of picomolar concentration of biomolecule in a liquid, the method must be very sensitive to ultra small changes. Whispering gallery mode (WGM) in dielectric resonator (DR) is well known to have high sensitivity and high *Q* factor and have been used for various sensing applications [11], [12], [13], [14], [15]. In the present study a composite single crystal sapphire DR with a disposable polycarbonate sample holding disk (SHD) is used to study the activity of pM concentration of insulin 25 millimolar (mM) Hepes buffer solution at 17 GHz microwave frequency for *WGE*_800_ mode. Hepes buffer with 35–378.78 pM concentration of insulin is used to measure permittivity and relaxation time.

## Experimental results and analysis

All the experimental observations presented here were carried out using Rohde & Schwarz ZVA 50 vector network analyser. Composite dielectric resonator comprised of *c*-axis oriented single crystal sapphire puck with dielectric constant 11.5, height 8 mm and diameter 20 mm and a SHD of thickness 1 mm and diameter 20 mm having a ring cavity of width 0.5 mm and 0.5 mm depth near the rim of the disk. In experimental measurement sapphire DR was found resonating at 17.59 GHz with *Q* value 86,122 at room temperature for *WGE*_800_ mode and composite DR was measured resonating at 17.5485 GHz with a 17,000 *Q* factor. Furthermore, calculated loss tangent of sapphire is 3.6 × 10^−6^ and for SHD is 0.00708. To study the activity of insulin pM concentrations in solution particular volumes of sample was loaded in 0.5 mm ring of SHD. For this various insulin solutions of desired concentration in the range found in human blood were prepared by diluting 10 mg/ml insulin solution (human recombinant) in 25 mM Hepes buffer solution, both were purchased from Sigma Aldrich. For present study different volumes of 0.2, 0.4, 0.8, 1.2 and 1.6 μL quantities with different concentration of insulin were analysed to study the dielectric relaxation and the time delay behaviour of the pM concentration solution of insulin maintained at 5°C during experimentation. Temperature was continuously monitored using digital probe type thermometer. In the present method, different concentrations of insulin solutions were loaded manually. The time taken for changing the sample and taking the observation was 15 s. These obtained results are discussed in detail as follows.

### Real and imaginary permittivity of insulin solution

Thought behind the present method is the change in dipole moment of solution, which affects the permittivity of the solution. This change directly relates to the state of activity of the present biological molecule in solution as discussed by Putz et al. [16]. Thus, by evaluating these properties it is possible to identify the activity period of any biological material *in vitro*. In the present study real and imaginary permittivity of different insulin concentrations in Hepes buffer solution was calculated by using the cavity perturbation method [11], [12], [17]. For calculating real and imaginary part of permittivity of solution we have used following equations presented in detail in Ref. [11] for used experimental arrangement:

(1) $$\frac{f_{\mathrm{SHD}}-f_{\mathrm{soln}.}}{f_{\mathrm{SHD}}}=A_{1}({ɛ}_{\mathrm{soln}.}^{\prime }-1)\frac{V_{\mathrm{soln}.}}{V_{\mathrm{DR}}+V_{\mathrm{SHD}}}$$

(2) $$\frac{1}{Q_{\mathrm{soln}.}}-\frac{1}{Q_{\mathrm{SHD}}}=A_{2}{ɛ}_{\mathrm{soln}.}^{\prime \prime }\frac{V_{\mathrm{soln}}}{V_{\mathrm{DR}}+V_{\mathrm{SHD}}}$$

Here, *ε*'_soln._ and *ε''*_soln._ represents real and imaginary permittivity of solution, *f, V* and *Q* represents the frequency volume and quality factor corresponding to its suffix. A_1_ and A_2_ are constants and calculated values for these are 0.01097 and 0.01903 respectively.

**Fig. 1:**
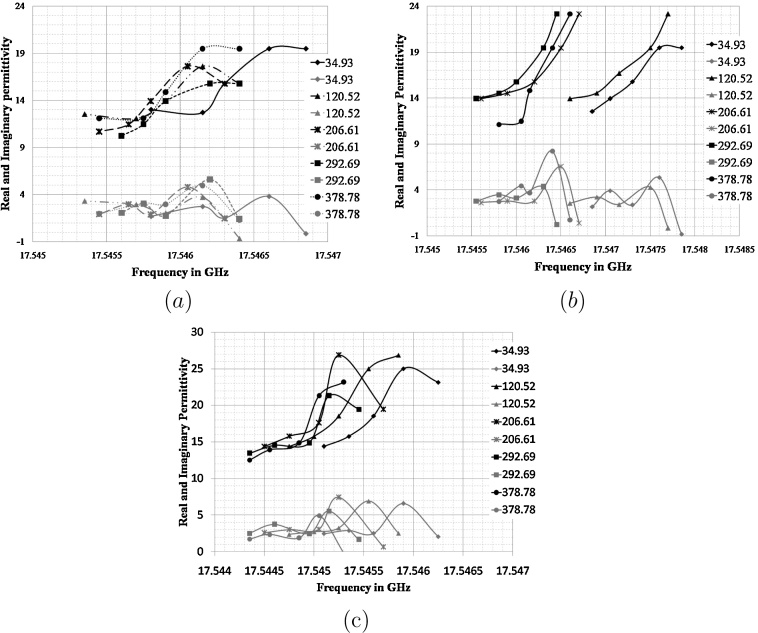
Real (black) and imaginary (grey) part of permittivity as a function of frequency for (a) fist day data, (b) second day data and (c) third day data.

**Fig. 2:**
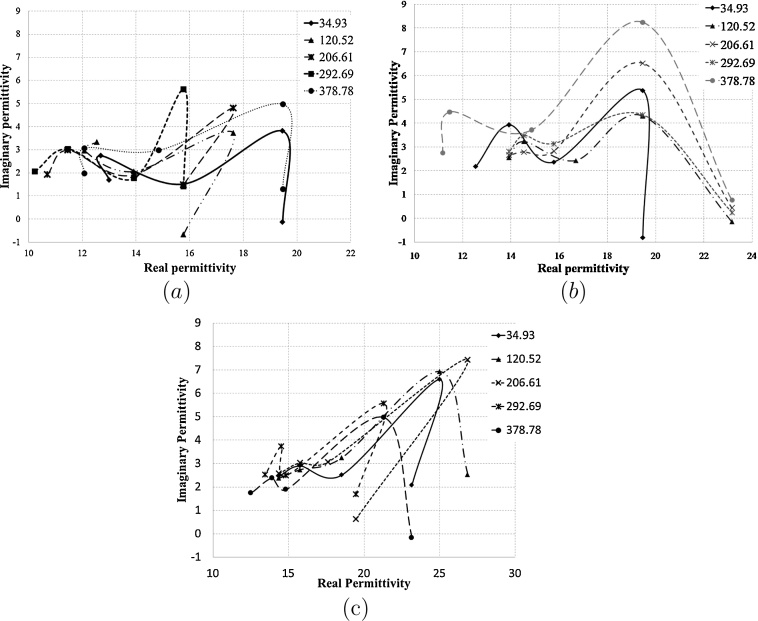
Cole–Cole type plot between real and imaginary part of permittivity for insulin solution for two days continuous measurement for (a) fist day data, (b) second day data and (c) third day data.

From Fig. 1 it is observed that when real permittivity is frequency dependent, losses (imaginary permittivity) are showing its maximum value, this indicates that results follow characteristic standard dispersion curve of biological tissues [11]. The real and imaginary part of permittivity show an increasing trend with frequency. On comparing the results obtained for three days on same sample sets (data shown in figure 1), it is observed that obtained values for real and imaginary permittivity for different concentration solution showing random and overlapping results with maximum 0.4 MHz shift in frequency for first day. Whereas for second day, it shows a consistent shift in frequency with increase in insulin pM concentration and observed maximum 1.3 MHz shift in frequency. For third day overlapping pattern with maximum shift of 0.8 MHz in frequency is observed which is clearly lower than second day data. Using the calculated values of real and imaginary permittivity with increase in resonant frequency a Cole–Cole graph is plotted in Fig. 2 for three days data.

From Fig. 2 it was observed that the data plotted in Fig. 2(b) show a consistent increase in covered area by plot with increase in concentration of insulin in solution. Whereas graph plotted with first and third day data exhibits random values with approximately the same covered area by plots with the increase in insulin concentration.

#### Dielectric relaxation study of insulin solution

From the measured values of permittivity for different concentration of same solution for three days it is observed that data taken on second day shows consistent behaviour. To further understand these results relaxation time for the same measured data was also calculated using the Debye equations. According to Debye dielectric theory, real and imaginary part of permittivity of solution is given by following equations:

(3) $${ɛ}_{\mathrm{soln}.}^{\prime }(\omega )={ɛ}_{\infty }+\frac{{ɛ}_{s}{ɛ}_{\infty }}{1+\omega^{2}\tau^{2}}$$

(4) $${ɛ}_{\mathrm{soln}.}^{\prime \prime }(\omega )=({ɛ}_{s}-{ɛ}_{\infty })\frac{\omega \tau }{1+\omega^{2}\tau^{2}}$$

Here, *ɛ*_*s*_ and *ɛ*_∞_ represent static permittivity, where, ${ɛ}_{\infty }=\lim_{\omega \to \infty }{ɛ}^{\prime }(\omega )$. Therefore, relaxation time of solution can be expressed as:

(5) $$\tau =\frac{1}{2\pi f}\times \frac{{ɛ}_{\mathrm{soln}}^{\prime \prime }}{{ɛ}_{\mathrm{soln}.}^{\prime }-{ɛ}_{\infty }}$$

where *ω* = 2*πf* represents the resonant frequency as the concentration of Insulin is very low thus *ɛ*_∞_ of solution can be taken same as for water. Thus, using *ɛ*_∞_ = 5.6 as found for water [14] at room temperature, relaxation time of solution is calculated by using Eq. (5).

**Fig. 3:**
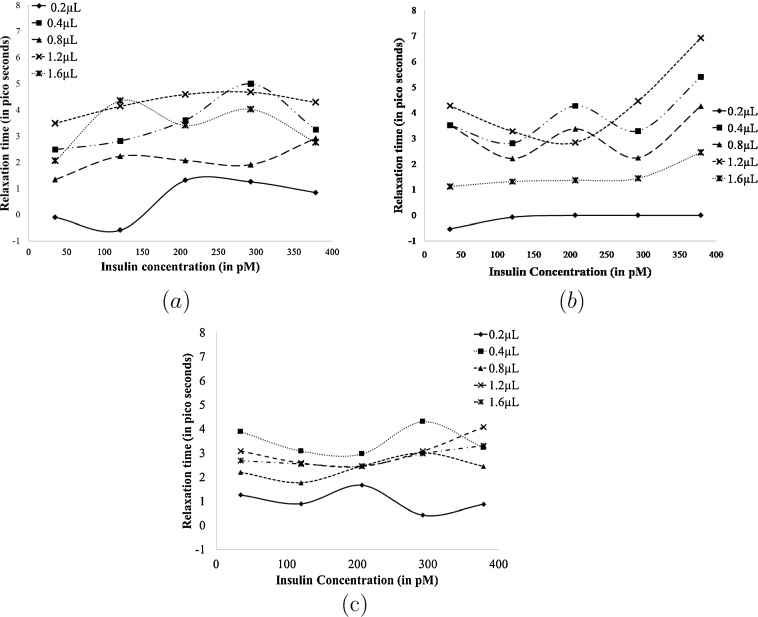
Relaxation time as a function of Insulin concentration for (a) fist day data, (b) second day data and (c) third day data.

Calculated relaxation time is plotted as a function of insulin pM concentration for three days data available shown in Fig. 3(a)–(c). From Fig. 3(b) relaxation time data for second day shows maximum change of 7 ps at a given volume for the range of insulin concentration loaded on SHD. Whereas, for first and third day data (in Fig. 3(a) and (c)) maximum change in relaxation time is less than 4 ps and shows random variation for different volumes loaded on SHD. Relaxation time is directly linked to the dipole moment of solution which depends on the biological activity of molecules present in solution [16] and molecules having a large dipole moment will show slow change in dipole orientation to the probing EM fields. From these observations it can be inferred that greater change in relaxation time on second day is due to the active insulin molecule and smaller change on first and third day is mainly due to less polar buffer solution.

## Discussion of results

*In vitro* time dependent degradation and stability study of proteins and insulin in medium have been carried out by various researchers to estimate the activity of biomolecule in solution with variation in other constituents of buffer solution and due to other environmental factors [6], [18], [19], [20], [21]. This experimental study investigates the decay pattern of insulin in Hepes buffer solution on diluting up to 35 pM concentration. Experimental measurement were spread over three days. On comparing the results for each day it is observed that on first day there is no consistent pattern whereas on the second day consistency was observed in measurement of varying concentrations of insulin. Therefore, it can be concluded that solution takes 24 h to stabilize. It has been reported that 24–37 h required for homogenization at 4°C [18] on diluting the protein solution. Results obtained here also show the same behaviour. Experimental data for third day with same samples of insulin solution starts showing random results with smaller variation in measured values as compared to second day data for all the concentrations of insulin. These results indicate that insulin solution became electrically less active for all concentration and degrading is started. This finding is consistent with the study presented by Putz et al. [16], that biological activity of the protein is directly depends on its dipole moment and it is known fact that dipole moment is linked with permittivity and relaxation time. But from results presented here for second day data it was observed that with the increase in insulin concentration there was an increase in permittivity and relaxation time. This indicates that now results are According to Basey et al. [22], in which it was found that for lower concentration of proteins in solution, ratio of change is permittivity to change in relaxation time remains constant i.e. they both are directly proportional. Furthermore, these results indicate that for second day data for Insulin solutions are in active state according to Putz et al. [16].

## Conclusion

Analysis presented in the present research work is to identify the activity of insulin solution with time over three days. Data is analysed by studying the change in permittivity, relaxation time and time delay at 17 GHz. From these analysed results it was observed that when an insulin solution of *pM* concentration is prepared in buffer solution it requires 24 h to saturate and stabilize. After 48 h it starts decaying even if the sample is maintained at 5°C. This method shows that permittivity, relaxation time, and time delay can be the indicative parameters to study the activity of biomolecules in different medium. This study is central in drug designing and delivery and this method can prove to be a quick method to understand the behaviour of biomolecules under different conditions.
